# PATTERNS OF ALCOHOL USE OVER TIME AMONG OLDER COUPLES: IMPLICATIONS FOR SELF-RATED HEALTH

**DOI:** 10.1093/geroni/igad104.2519

**Published:** 2023-12-21

**Authors:** Angela Turkelson, Kira Birditt, Courtney Polenick, James Cranford, Fred Blow

**Affiliations:** University of Michigan: Institute for Social Research, Ann Arbor, Michigan, United States; Institute for Social Research, University of Michigan, Ann Arbor, Michigan, United States; University of Michigan, Ann Arbor, Michigan, United States; University of Michigan, Ann Arbor, Michigan, United States; University of Michigan, Ann Arbor, Michigan, United States

## Abstract

Alcohol use in older adults is increasing which can have implications for health outcomes. Research has examined longitudinal patterns of drinking for individuals over time, but not couples. Couples often engage in concordant drinking behaviors but it is unknown if couples become more or less concordant or discordant in their drinking over time or how increased concordance or discordance influences health. The purpose of this study was to examine patterns of alcohol use over time among older couples and their links with self-reported health. A total of 8,570 husbands and wives completed the main interview of the Health and Retirement Study (HRS) for at least three consecutive biennial waves between 1996 and 2016. Latent class growth analysis revealed five trajectories of couple drinking: 1. Concordant - husband and wife light (n = 6398, 75%); 2. Concordant - husband and wife light/moderate (n = 781, 9%); 3. Concordant - husband and wife moderate (n = 296, 3.5%); 4. Discordant - husband heavy and wife light (n = 293, 3.5%); 5. Discordant - husband moderate and wife light (n = 802, 9%). Discordant couples and concordant light couples reported worse self-rated health over time compared with concordant light/moderate and concordant moderate couples. The most common pattern of drinking was concordant drinking with husbands showing light decreasing drinking and wives showing light stable drinking over time. Findings are consistent with literature and suggest that discordant drinking couples have poorer health.

